# Case Report: Long-term disease-free survival in an advanced hepatocellular carcinoma patient: an exceptional response to PD-1 inhibitor therapy

**DOI:** 10.3389/fimmu.2025.1677724

**Published:** 2025-11-11

**Authors:** Qinliang Fang, Shuqi Yu, Yu Xiong, Yibin Zhang, Xiaomin Wang, Jianyin Zhou, Fuqiang Wang, Zhenyu Yin

**Affiliations:** 1 Department of Hepatobiliary Surgery, Zhongshan Hospital of Xiamen University, School of Medicine, Xiamen University, Xiamen, Fujian, China; 2 Xiamen Translational Medical Key Laboratory of Digestive System Tumor, Fujian Provincial Key Laboratory of Chronic Liver Disease and Hepatocellular Carcinoma, Xiamen University, Xiamen, Fujian, China; 3 Department of Pathology, Zhongshan Hospital of Xiamen University, School of Medicine, Xiamen University, Xiamen, Fujian, China

**Keywords:** hepatocellular carcinoma, PD-1 inhibitor, disease-free survival, pembrolizumab, follow-up

## Abstract

**Introduction:**

Hepatocellular carcinoma (HCC) is a leading cause of cancer-related death, with most patients diagnosed at advanced stages, often precluding surgical resection. Recently, immune checkpoint inhibitors, particularly PD-1 inhibitors, have emerged as promising therapies, though long-term disease-free survival (DFS) remains rare. We report a case of an advanced HCC patient who achieved complete remission (CR) after just four cycles of reduced-dose pembrolizumab and maintained a disease-free status for more than seven years.

**Case report:**

A 55-year-old male with chronic hepatitis B and alcohol-related liver disease presented with a ruptured HCC. After initial treatments, including surgery and chemotherapy, the patient was started on reduced-dose pembrolizumab (100 mg). After four cycles, the patient achieved CR.

**Conclusion:**

This case highlights the potential for long-term survival with reduced-dose PD-1 inhibitor therapy in advanced HCC. The patient’s exceptional response provides important insights into the role of personalized dosing, immune checkpoint inhibitors, and the management of immune-related adverse events.

## Introduction

Hepatocellular carcinoma (HCC) is one of the most common and lethal malignancies worldwide, with more than 850,000 new cases reported annually (1). Despite advances in screening and early detection, most patients are diagnosed at an advanced stage, losing the opportunity for curative surgery, or experience recurrence after surgery. For these patients, systemic and locoregional therapies remain the primary treatment options.

In recent years, multimodal treatment strategies combining transarterial chemoembolization (TACE), hepatic arterial infusion chemotherapy (HAIC), molecular targeted therapy, and immunotherapy have significantly changed the treatment landscape of advanced HCC (2). Tyrosine kinase inhibitors (TKIs), including sorafenib and lenvatinib, have long been the standard first-line systemic therapy for advanced HCC (3). However, despite providing survival benefits, TKIs alone rarely lead to CR.

The emergence of immune checkpoint inhibitors (ICIs), particularly programmed death-1 (PD-1) inhibitors, has revolutionized the treatment of advanced HCC. Landmark trials, such as the IMbrave150 study, demonstrated that PD-1/PD-L1 blockade in combination with anti-angiogenic therapy (e.g., atezolizumab + bevacizumab) significantly improves overall survival (OS) and progression-free survival (PFS) compared to sorafenib alone (4). However, while a subset of patients exhibit durable responses, the overall objective response rate (ORR) remains low (20–30%), and long-term disease-free survival is rarely reported (5).

Predicting which patients will derive long-term benefits from PD-1 blockade remains a major challenge. Emerging research suggests that biomarkers such as PD-L1 expression, tumor mutational burden (TMB), and immune microenvironment characteristics may influence treatment response (6). However, the optimal duration of PD-1 inhibitor therapy, particularly in patients who achieve CR, remains unclear. While some studies suggest that continuing therapy for up to 2 years improves outcomes, others indicate that early discontinuation does not always lead to disease relapse (7).

Here, we report a remarkable case of an advanced HCC patient who achieved CR after only four doses of PD-1 inhibitor therapy and remained disease-free for over seven years. This case provides valuable insights into the potential for long-term survival with PD-1 inhibitors, the role of personalized dosing strategies, and the need for predictive biomarkers to guide treatment selection.

## Case report

### Patient background and diagnosis

**Figure 1:**
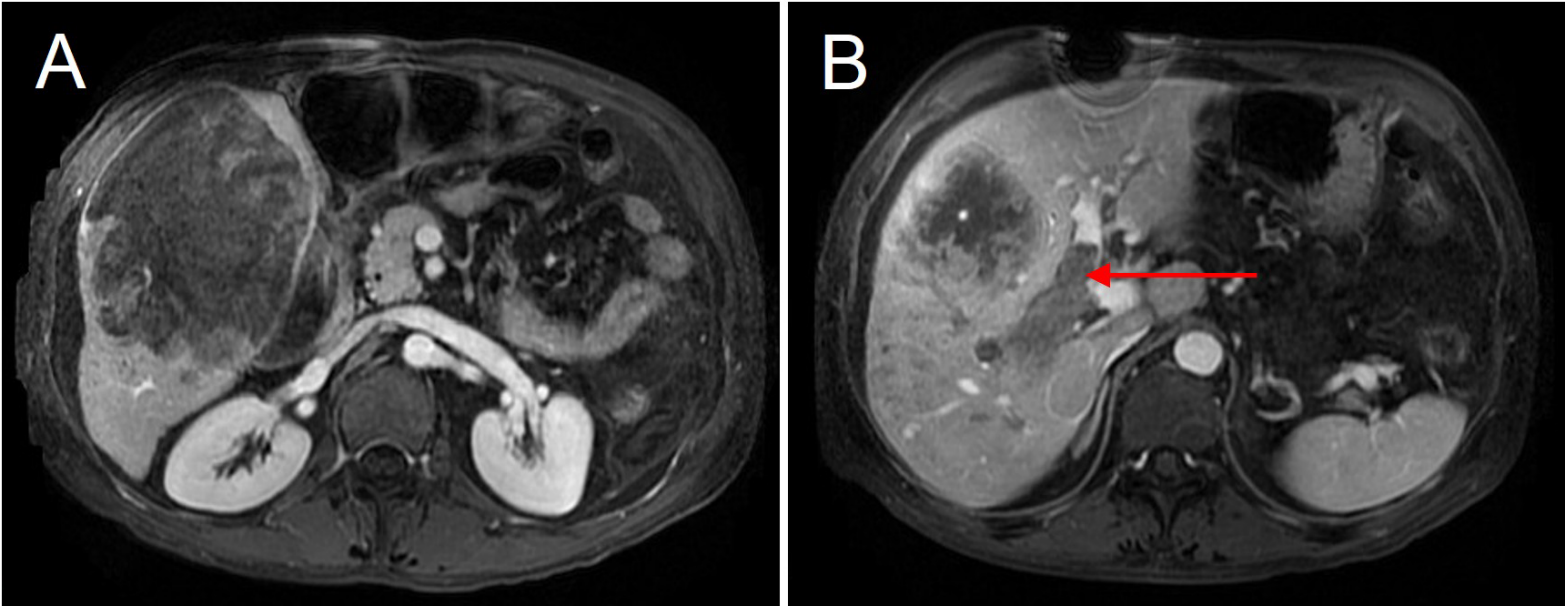
Preoperative MRI showing a giant hepatocellular carcinoma in the right hepatic lobe. **(A)** The tumor measures approximately 15 cm × 10 cm in size. **(B)** The right portal vein is completely filled with tumor thrombus (red arrow), indicating portal vein invasion by the tumor.

A 55-year-old male with a history of chronic hepatitis B infection and alcohol-related cirrhosis was admitted to the hospital due to acute abdominal pain and hemodynamic instability. In addition, twelve years earlier, the patient underwent surgical repair for a perforated gastric ulcer and recovered well after the operation. Upon this admission, Contrast-enhanced MRI revealed a ruptured hepatocellular carcinoma (HCC) in the right hepatic lobe, measuring approximately 150 × 110 × 100mm, accompanied by portal vein tumor thrombus (PVTT, Cheng’s type II) (**Figure 1**).

Laboratory tests showed significantly elevated alpha-fetoprotein (AFP) levels (1147 ng/mL) and mild liver dysfunction (Child-Pugh A/B). The patient was classified as Barcelona Clinic Liver Cancer (BCLC) stage C and China Liver Cancer (CNLC) stage IIIa.

Upon admission, the patient was initiated on oral antiviral therapy for hepatitis B virus (HBV) suppression to prevent HBV reactivation during subsequent treatments.

### Emergency intervention, surgery, and subsequent treatments

Emergency transarterial embolization (TAE) was performed to control bleeding and stabilize the patient’s hemodynamic condition. (**Supplementary Material 1**). After hemodynamic stabilization had been achieved, a multidisciplinary team (MDT) discussion was conducted, and the decision was made to proceed with right hemihepatectomy, cholecystectomy, and PVTT thrombectomy.

**Figure 2:**
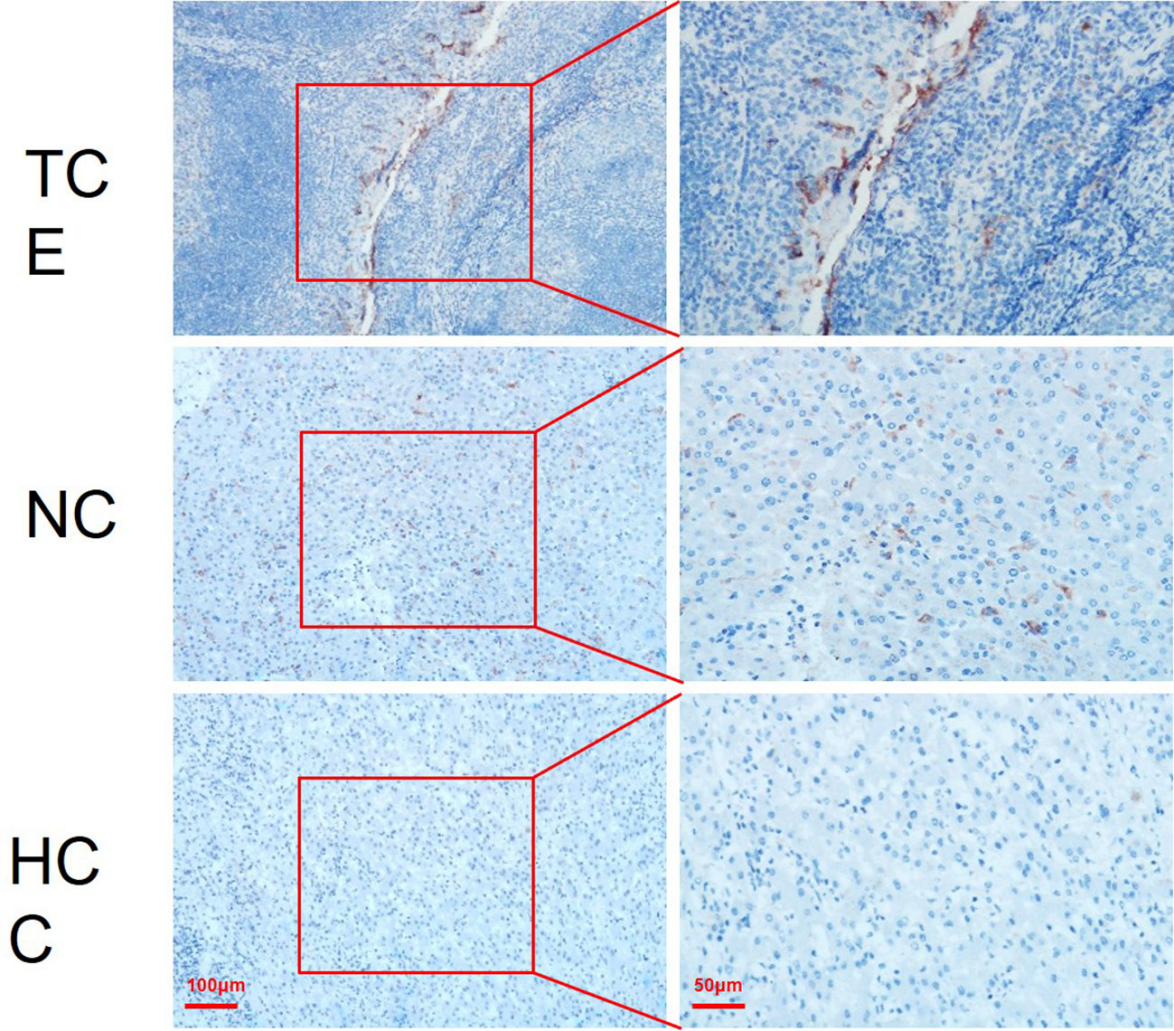
PD-L1 expression in tonsillar crypt epithelium, adjacent liver tissue, and hepatocellular carcinoma. **(A)** Tonsillar Crypt Epithelium (TCE) as a positive control, demonstrating strong membranous and cytoplasmic PD-L1 expression, consistent with the known high expression in TCE. **(B)** Adjacent non-tumorous liver tissue from a hepatocellular carcinoma (HCC) patient, showing positive PD-L1 expression primarily localized to immune cells. **(C)** Hepatocellular carcinoma tissue, where PD-L1 staining is negative, with almost no detectable expression in tumor cells. Red boxes indicate the magnified areas, with corresponding high-power images to the right. (Bar = 100 μm or 50 μm).

Intraoperative findings confirmed a large, ruptured tumor with approximately 1000 mL of hemoperitoneum. The resected specimen exhibited satellite nodules and right hepatic vein tumor thrombus. Postoperative pathology revealed moderately differentiated hepatocellular carcinoma with microscopic vascular invasion, and cirrhotic changes in the surrounding liver tissue. PD-L1 immunohistochemical staining was performed on both cancerous and adjacent non-tumorous tissues. The analysis demonstrated positive PD-L1 expression in the adjacent non-tumorous tissue. In contrast, PD-L1 staining in the hepatocellular carcinoma tissue was almost negative, with minimal expression in tumor cells (**Figure 2**).

**Figure 3:**
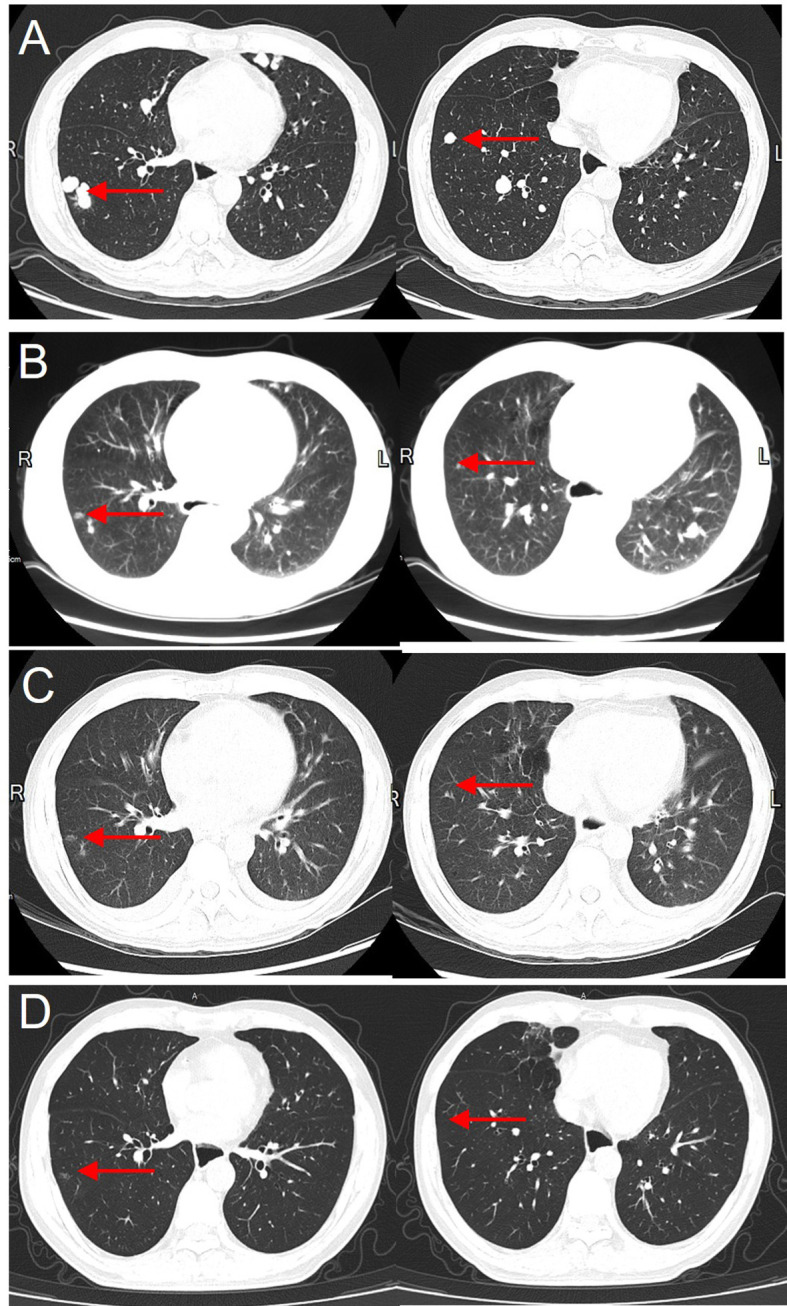
Gradual disappearance of lung metastases following PD-1 inhibitor therapy, with no recurrence after 7 years. **(A)** Pre-treatment CT showing the largest lung metastasis with a diameter of 1.5 cm before starting PD-1 inhibitor therapy. **(B)** Two months after PD-1 inhibitor therapy, the lung metastasis has significantly reduced in size. **(C)** Four months after treatment, the lung metastasis is nearly completely resolved. **(D)** Seven years after PD-1 inhibitor therapy, follow-up chest CT shows no recurrence of lung metastases.

At postoperative week 2, the patient underwent prophylactic transarterial chemoembolization (TACE). Systemic therapy with sorafenib was initiated but demonstrated poor tumor control, and intrahepatic recurrence with pulmonary metastases was detected at six months postoperatively (**Figure 3**). Thereafter, sorafenib was discontinued, and the patient underwent additional TACE (**Supplementary Material 2**) and FOLFOX4 chemotherapy. At postoperative month 10, thymosin α1 was introduced as an adjuvant therapy; however, The disease exhibited continuous progression despite treatment.

### Introduction of PD-1 inhibitor therapy and tumor response

At 15 months postoperatively, the patient was initiated on pembrolizumab (PD-1 inhibitor) therapy. Importantly, the patient received a reduced dose of 100 mg per session, rather than the standard weight-based dose.

**Figure 4:**
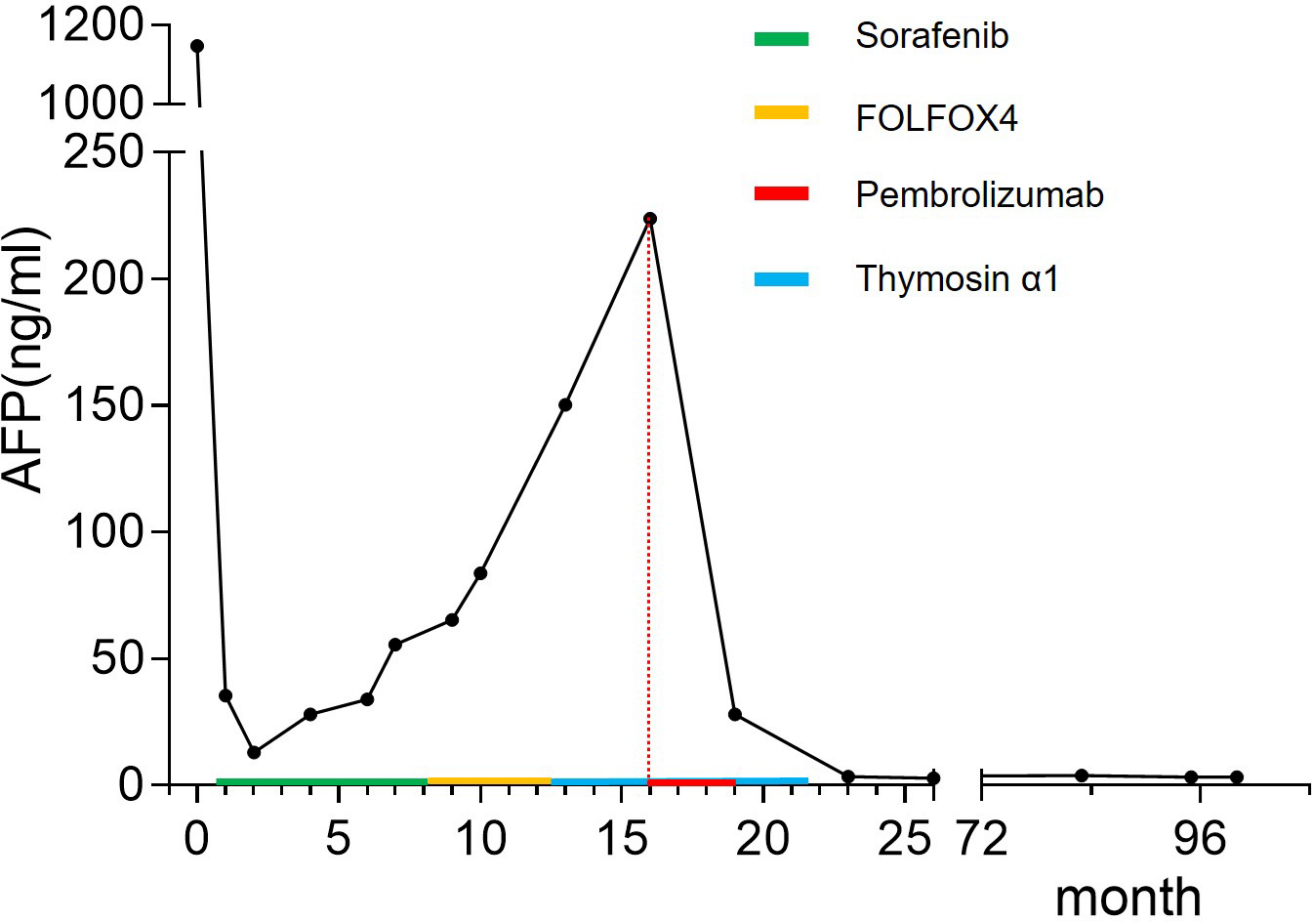
Changes in alpha-fetoprotein (AFP) levels throughout the treatment course. The patient’s AFP levels initially decreased from a peak of 1147 ng/mL postoperatively to a low of 13.07 ng/mL. Despite treatment with sorafenib, FOLFOX4, and thymosin α1, AFP levels gradually increased and reached 223.9 ng/mL prior to the initiation of pembrolizumab therapy. After receiving pembrolizumab, AFP levels rapidly declined, and by five months post-treatment, AFP returned to normal levels.

After two cycles, follow-up imaging revealed evident tumor regression accompanied by a substantial decline in AFP levels (**Figure 4**), indicating a strong anti-tumor response. Following four cycles of PD-1 inhibitor therapy, the patient achieved complete remission (CR) and subsequently discontinued treatment entirely. Following the third dose, the patient developed hypothyroidism, gastrointestinal toxicity (severe diarrhea), poor appetite, and psychiatric symptoms including hallucinations. These symptoms markedly improved following glucocorticoid therapy.

Subsequent follow-up imaging confirmed the absence of tumor recurrence or metastasis, AFP levels returned to normal, and PET-CT scans confirmed a complete metabolic response (CMR).

### Long-term follow-up

As of the latest follow-up (7 years post-treatment), the patient remains in sustained complete remission (CR), with no evidence of tumor recurrence or metastasis. This case represents one of the longest reported disease-free survival periods in an advanced HCC patient achieving CR after only four cycles of PD-1 inhibitor therapy (**Supplementary Material 3**).

The patient has maintained good general health with stable liver function and no notable long-term adverse effects associated with immunotherapy. Regular follow-up is being conducted to monitor for recurrence or late adverse events, and further updates on long-term survival outcomes will be reported.

## Discussion

This case provides valuable insight into the exceptional long-term response to PD-1 inhibition in advanced hepatocellular carcinoma (HCC), even with a reduced-dose regimen. The remarkable durability of the response over seven years makes this case an excellent opportunity to discuss several aspects of immunotherapy for HCC, including the mechanisms behind the exceptional response, the synergistic effects between thymosin α1 and PD-1 inhibitors, and the management of immune-related adverse events (irAEs). Although there are currently cases of long-term survival under comprehensive treatments such as targeted therapy combined with immunotherapy, there have been no reports of long-term disease-free survival in patients who achieved complete remission (CR) after short-term monotherapy with low-dose PD-1 inhibitors.

1. The patient’s exceptional response to a reduced dose of pembrolizumab (100 mg per session) instead of the standard weight-based regimen can be attributed to several key factors. First, the patient had a history of chronic hepatitis B, with a small liver remnant and poor liver reserve, which raised concerns about liver function and drug metabolism. Higher doses of immune checkpoint inhibitors (ICIs) could potentially lead to immune-related hepatitis and further liver damage, making the reduced dose a safer choice. Second, the patient’s performance status had deteriorated due to multiple treatments, necessitating a cautious approach to minimize toxicity while still ensuring effective tumor control. This suggests that reduced-dose PD-1 inhibitors could be a viable strategy for patients with compromised hepatic function and lower performance status. Lastly, the postoperative histopathological analysis did not show strong PD-L1 expression in the tumor, possibly due to antigen degradation during specimen preservation. This absence of detectable PD-L1 suggests that the patient’s exceptional response to immunotherapy might be driven by alternative, yet-to-be-identified immunologic mechanisms. Further molecular investigations, including next-generation sequencing (NGS) and tumor mutational burden (TMB) analysis, are warranted to elucidate the biological mechanisms underlying this exceptional response. Although the treatment dose was reduced, the patient exhibited a marked antitumor response, highlighting the need for further investigation into optimal dosing strategies for immune checkpoint inhibitors, especially in individuals who develop severe PD-1–related immune adverse events.
2. In this case, the use of thymosin α1, a thymic peptide known for its ability to enhance immune function, played a significant role in the treatment of hepatocellular carcinoma (HCC). Thymosin α1 works by stimulating T-cell proliferation, enhancing dendritic cell activity, and promoting cytokine release. When combined with PD-1 inhibitors, it can boost T-cell activation and cytokine production, leading to a more robust anti-tumor immune response. Specifically, thymosin α1 has been shown to restore T-cell function in patients with immunosuppressive conditions like cancer and chronic viral infections, helping activate cytotoxic T-cells and thereby enhancing the efficacy of PD-1 inhibitors (8, 9). Furthermore, thymosin α1 may improve immune memory, which is essential for preventing recurrence after the discontinuation of treatment. By promoting long-lived, activated T-cells, it aids in maintaining immune surveillance against residual tumor cells even after stopping PD-1 therapy (10). The combination of thymosin α1 and PD-1 inhibitors also appears to counteract immune exhaustion and reduce the immunosuppressive factors in the tumor microenvironment, which may further enhance the anti-tumor immune response and improve clinical outcomes. This synergistic approach could represent a promising strategy for treating advanced HCC by bolstering the immune system’s ability to recognize and eliminate cancer cells.
3. Immune checkpoint inhibitors, while highly effective, can also lead to a range of immune-related adverse events (irAEs), as seen in this case where the patient developed severe psychiatric symptoms (hallucinations), gastrointestinal toxicity (severe diarrhea), and thyroid dysfunction (hypothyroidism). These irAEs were managed with a combination of therapies. The psychiatric symptoms, including hallucinations, which are rare but recognized side effects of PD-1 inhibitors, were thought to be caused by autoimmune inflammation of the central nervous system. These manifestations were successfully managed with glucocorticoid therapy, emphasizing the importance of early recognition and prompt intervention (11). The patient also experienced severe diarrhea, a well-known gastrointestinal side effect of PD-1 inhibitors, typically caused by T-cell-mediated intestinal inflammation. This severe diarrhea was managed effectively with glucocorticoid therapy, underscoring the necessity of prompt intervention to prevent serious complications like intestinal perforation or dehydration (12). Additionally, the patient developed hypothyroidism due to immune-mediated thyroiditis, a known endocrine side effect of PD-1 inhibitors. Thyroxine supplementation was used to manage this, leading to the normalization of thyroid function and the resolution of related symptoms (13). These side effects suggest that patients experiencing strong anti-tumor effects from PD-1 inhibitors may also face more pronounced adverse events, potentially due to dynamic changes in proliferating T-cells. Early detection and management of these irAEs are crucial for improving patient outcomes and minimizing long-term complications (14).
4. The optimal duration of PD-1 inhibitor therapy for patients who achieve complete remission (CR) is still a subject of debate. While some studies suggest continuing therapy for up to two years, this patient discontinued treatment after just four doses and has remained in sustained remission for seven years. Several factors should be considered when deciding whether to stop PD-1 inhibitors. Prolonged exposure to these inhibitors increases the risk of immune-related toxicity, and continuing therapy beyond the point of complete remission may not offer additional benefits (15). On the other hand, early discontinuation does not always lead to recurrence, especially in patients who demonstrate a strong immune response and achieve CR (16). Furthermore, immune memory may be sufficient to maintain tumor surveillance even after stopping therapy, indicating that immune checkpoint inhibitors may have long-lasting effects in some patients (17). This case offers clinical evidence suggesting that early discontinuation of PD-1 inhibitors may not compromise long-term outcomes, suggesting that immune memory plays a role in maintaining disease-free survival. This finding underscores the importance of conducting further investigations to identify predictive biomarkers, determine optimal dosing regimens, and define individualized treatment durations for PD-1 inhibitors in hepatocellular carcinoma. In addition, given the patient’s history of alcohol consumption and the inter-individual variability in immune and genetic factors, this case represents a unique clinical scenario, and caution should be exercised when generalizing these findings to the broader HCC population.

## Conclusion

This case report highlights the extraordinary long-term disease-free survival (DFS) achieved in an advanced hepatocellular carcinoma (HCC) patient treated with reduced-dose PD-1 inhibitor therapy. The patient’s exceptional therapeutic response, with seven years of disease-free survival, emphasizes the need for further investigation into the mechanisms behind such an exceptional outcome. While the reduced dose was a unique aspect of this treatment, the main focus is on understanding the reasons behind the patient’s highly favorable response, not on promoting the advantages of dose reduction. Additionally, this case emphasizes the relationship between strong anti-tumor responses to PD-1 inhibitors and the potential for increased immune-related adverse events (irAEs). In this case, the patient’s extraordinary response was accompanied by an increase in the severity of immune side effects, highlighting the importance of monitoring and managing these reactions carefully. This case exemplifies that short-course PD-1 inhibitor therapy can achieve sustained long-term disease-free survival in advanced HCC, a result that has yet to be reported elsewhere globally. This report enhances our understanding of the potential role of immunotherapy in improving long-term survival outcomes in advanced hepatocellular carcinoma and underscores the necessity for continued research to refine therapeutic strategies and elucidate the underlying mechanisms of such exceptional clinical responses.

## Patient perspective

The patient shared his emotional journey in battling hepatocellular carcinoma. The initial diagnosis of a ruptured liver tumor and the subsequent emergency treatments brought great fear and uncertainty to him and his family. Multiple interventions, including surgery, TACE, chemotherapy, and immunomodulatory therapy, were exhausting and often accompanied by side effects such as fatigue, loss of appetite, and insomnia. However, after starting pembrolizumab treatment, despite experiencing several severe adverse effects, the rapid control of the tumor greatly encouraged him to overcome difficulties and ultimately achieve complete remission. The disappearance of the tumor and long-term stability during follow-up reignited his confidence and optimism about life. He expressed deep gratitude to the medical team for their professional care, timely interventions, and continuous support throughout the entire treatment process. The patient emphasized that this experience not only saved his life but also strengthened his belief in science and personalized medicine.

## Data Availability

The original contributions presented in the study are included in the article/**Supplementary Material**. Further inquiries can be directed to the corresponding authors.

## Supplementary material

Supplementary Material 1 Diagnosis of ruptured hepatocellular carcinoma and emergency transarterial embolization for hemostasis. **(A)** Contrast-enhanced CT at admission revealed a large mass in the right hepatic lobe, suspicious for hepatocellular carcinoma. **(B, C)** Massive ascites and hemoperitoneum were seen in the abdominal and pelvic cavities (red arrows), indicating tumor rupture with intraperitoneal bleeding. **(D, E)** Emergency transarterial embolization (TAE) was performed. Hepatic arteriography showed a hypervascular tumor with focal contrast extravasation. A 2.5F microcatheter was superselectively advanced into the tumor-feeding artery (red arrows), and embolization was achieved using 700–1000 μm gelatin sponge particles. **(F)** Post-embolization angiography demonstrated satisfactory occlusion of the target vessel with marked reduction in tumor vascular staining.

Supplementary Material 2 Recurrence of hepatocellular carcinoma six months after liver resection and treatment with transarterial chemoembolization (TACE). **(A)** Contrast-enhanced CT in the arterial phase showing a recurrent tumor at the liver resection margin (red arrow). **(B)** CT in the venous phase confirming the recurrence. **(C, D)** Multiple lung metastases identified on CT (red arrows). **(E)** Angiography reveals a hypervascular nodule at the original liver resection margin, indicating tumor recurrence. A microcatheter was superselectively advanced into the tumor-feeding artery, and chemoembolization was performed.

Supplementary Material 3 Timeline. Clinical history and therapeutic interventions of the case.
