# Supplementary figures and images for: Case Report: Long-term disease-free survival in an advanced hepatocellular carcinoma patient: an exceptional response to PD-1 inhibitor therapy
Source: Front Immunol. 2025 Nov 11;16:1677724. doi: 10.3389/fimmu.2025.1677724 (PMC12643963; doi:10.3389/fimmu.2025.1677724)

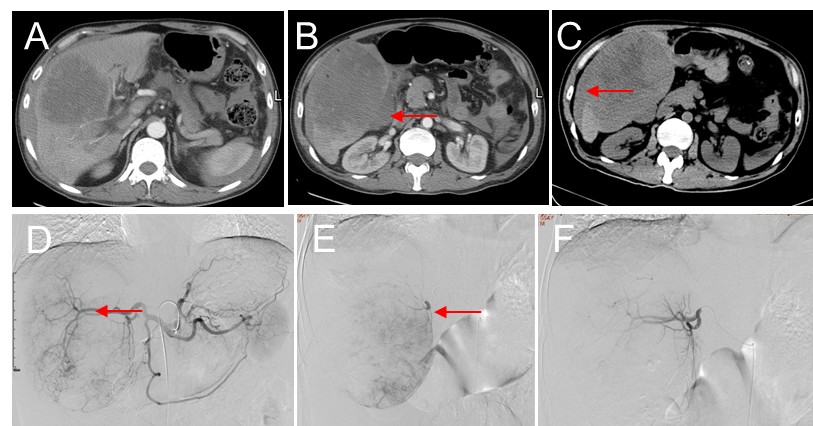

Supplement: Supplementary Material 1 — Diagnosis of ruptured hepatocellular carcinoma and emergency transarterial embolization for hemostasis. (A) Contrast-enhanced CT at admission revealed a large mass in the right hepatic lobe, suspicious for hepatocellular carcinoma. (B, C) Massive ascites and hemoperitoneum were seen in the abdominal and pelvic cavities (red arrows), indicating tumor rupture with intraperitoneal bleeding. (D, E) Emergency transarterial embolization (TAE) was performed. Hepatic arteriography showed a hypervascular tumor with focal contrast extravasation. A 2.5F microcatheter was superselectively advanced into the tumor-feeding artery (red arrows), and embolization was achieved using 700–1000 μm gelatin sponge particles. (F) Post-embolization angiography demonstrated satisfactory occlusion of the target vessel with marked reduction in tumor vascular staining. [file Image1.jpeg]

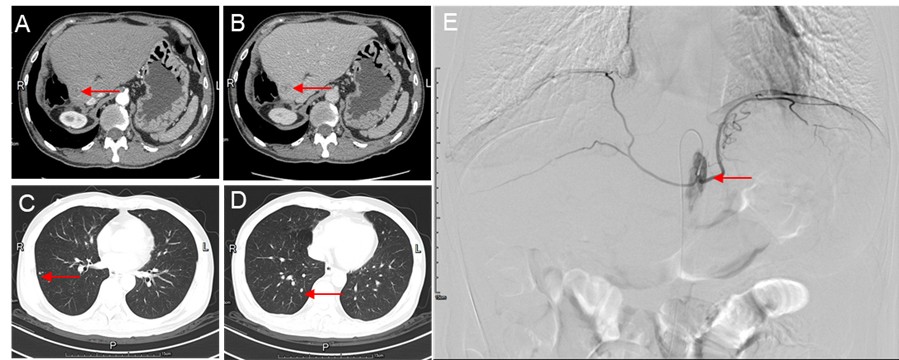

Supplement: Supplementary Material 2 — Recurrence of hepatocellular carcinoma six months after liver resection and treatment with transarterial chemoembolization (TACE). (A) Contrast-enhanced CT in the arterial phase showing a recurrent tumor at the liver resection margin (red arrow). (B) CT in the venous phase confirming the recurrence. (C, D) Multiple lung metastases identified on CT (red arrows). (E) Angiography reveals a hypervascular nodule at the original liver resection margin, indicating tumor recurrence. A microcatheter was superselectively advanced into the tumor-feeding artery, and chemoembolization was performed. [file Image2.jpeg]

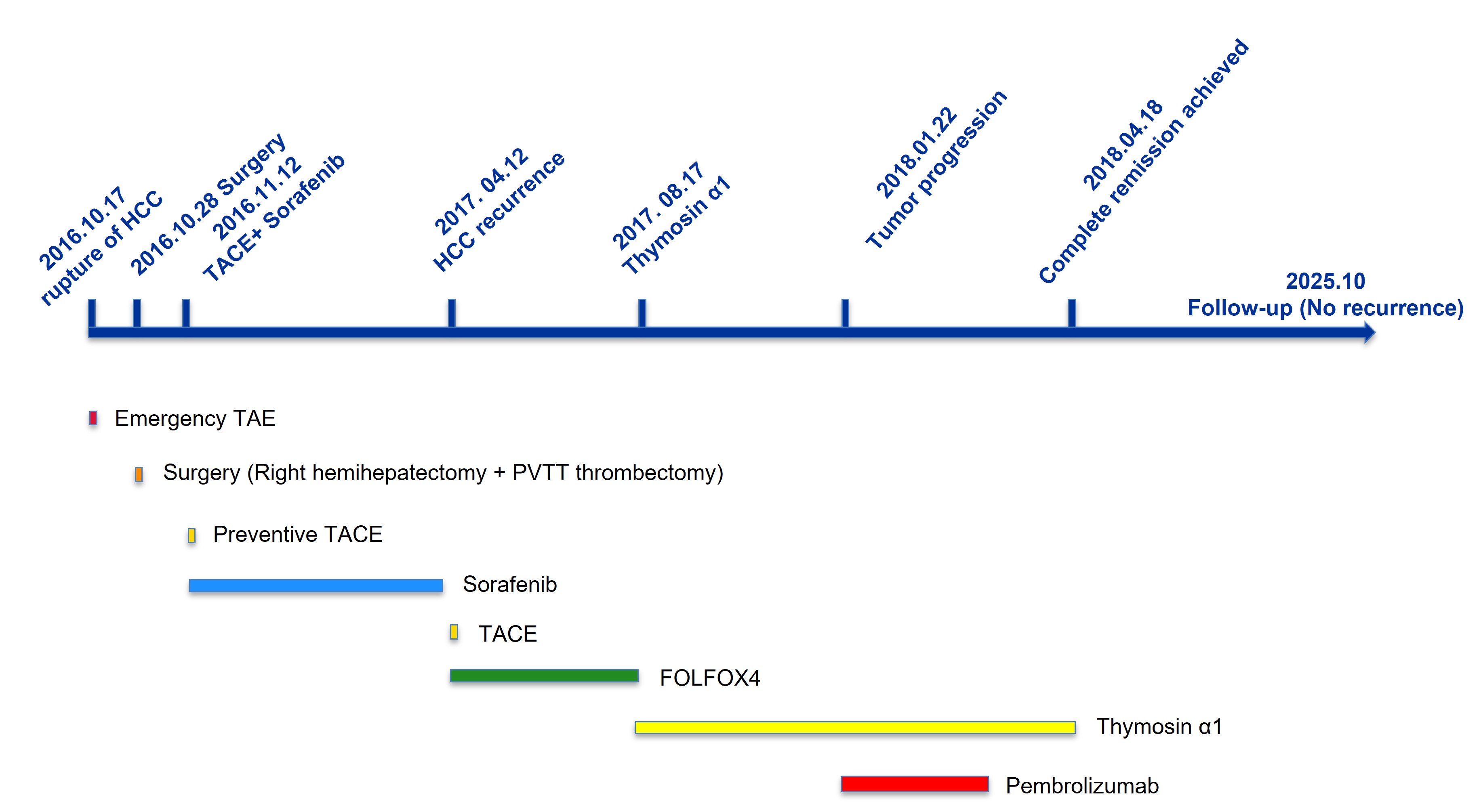

Supplement: Supplementary Material 3 — Timeline. Clinical history and therapeutic interventions of the case. [file Image3.jpeg]
